# LATE-LIFE OUTCOMES OF FEELINGS OF BELONGING THROUGHOUT THE LIFE COURSE

**DOI:** 10.1093/geroni/igae098.3422

**Published:** 2024-12-31

**Authors:** Emily Briggs, Jacqui Smith

**Affiliations:** University of Michigan, Ann Arbor, Michigan, United States; University of Michigan, Ann Arbor, Michigan, United States

## Abstract

Belonging is a fundamental human need that encourages fortitude throughout the life course. In the literature, belonging has been studied primarily from two perspectives: place attachment (Chawla, 1992, Scannell, 2021) and belonging within a social group (Allen, 2020). Less research, however, has been conducted about how belonging is experienced by the individual – self-perceptions of belonging. Using data from the Health and Retirement Study Life History Mail Survey (N=3,091, M Age =70.48 [50-98]) we investigated retrospective self-reports of belonging from three life stages, childhood, adolescence and midlife, as predictors of belonging and activity engagement in late life. First, we conducted linear regressions controlling for sociodemographic variables (gender, age, race, marital status). This revealed that self-reported feelings of belonging in childhood (β=.11, p<.001), adolescence (β=.13, p<.001), and midlife (β=.23, p<.001) were significantly predictive of heightened feelings of belonging in late life (Adj R2=.043,.051,.068). Second, we assessed self-reported belonging in childhood, adolescence, midlife and late life as predictors of late life engagement. These analysis revealed that belonging in childhood (β=.06, p<.001), midlife (β=.15, p<.001), and late life (β=.13, p<.001) were significantly predictive of heightened activity engagement in late life (Adj R2=.026,.037,.040). Overall this study demonstrates the importance of assessing how individuals think about their belonging retrospectively throughout their lifespan as a predictor of subsequent late-life belonging and activity engagement– both protective factors as a person ages.

